# Using the WHO-AIMS to inform development of mental health systems: the case study of Makueni County, Kenya

**DOI:** 10.1186/s12913-020-4906-3

**Published:** 2020-01-20

**Authors:** Victoria N. Mutiso, Christine W. Musyimi, Isaiah Gitonga, Albert Tele, Romaisa Pervez, Tahilia J. Rebello, Kathleen M. Pike, David M. Ndetei

**Affiliations:** 1 Africa Mental Health Research and Training Foundation, Mawensi Road, Off Elgon Road, Mawensi Gardens, P.O.BOX 48423-00100, Nairobi, Kenya; 2 Department of Epidemiology and Biostatistics, Schulich School of Medicine and Dentistry, Western University, 1151 Richmond St, London, Ontario N6A 5C1 Canada; 3 Columbia University Global Mental Health Program, 1051 Riverside Drive, New York, NY 1003 USA; 4 Department of Psychiatry, University of Nairobi, P. O. Box 30197 - 00100, Nairobi, Kenya

**Keywords:** Mental health systems, WHO-AIMS, LMIC, Makueni, Kenya

## Abstract

**Background:**

In order to develop a context appropriate in mental health system, there is a need to document relevant existing resources and practices with a view of identifying existing gaps, challenges and opportunities at baseline for purposes of future monitoring and evaluation of emerging systems. The World Health Organization Assessments Instrument for Mental Health Systems (WHO-AIMS) was developed as a suitable tool for this purpose. Our overall objective of this study, around which research questions and specific aims were formulated, was to establish a baseline on mental health system as at the time of the study, at Makueni County in Kenya, using the WHO-AIMS.

**Methods:**

To achieve our overall objective, answer our research questions and achieve specific aims, we conducted a mixed methods approach in which we did an audit of DHIS records and county official records, and conducted qualitative interviews with the various officers to establish the fidelity of the data according to their views. The records data was processed via the prescribed WHO-Aims 2.2 excel spreadsheet while the qualitative data was analyzed thematically. This was guided by the six domains stipulated in the WHO AIMS.

**Results:**

We found that at the time point of the study, there were no operational governance, policy or administrative structures specific to mental health, despite recognition by the County Government of the importance of mental health. The identified interviewees and policy makers were cooperative and participatory in identifying the gaps, barriers and potential solutions to those barriers. The main barriers and gaps were human and financial resources and low prioritization of mental health in comparison to physical conditions. The solutions lay in bridging of the gaps and addressing the barriers.

**Conclusion:**

There is a need to address the identified gaps and barriers and follow up on solutions suggested at the time of the study, if a functional mental health system is to be achieved at Makueni County.

## Background

High Income Countries (HIC) have adopted effective systems and approaches to mental health through rigorous studies [1, 2]. However, unlike Low and Middle Income Countries (LMIC), HIC have the resources and logistics to achieve this. Several LMIC have made attempts to address mental health systems, from different perspectives. In Brazil, efforts have been made to: (1) develop a mental health system to promote respect for the rights of people with mental disorders, (2) gradually replace psychiatric beds with community-based and primary healthcare mental health services, and (3) promote training and financial support to change the mental health care paradigm [3]. In South East Asia, seven out of the 11 countries have made use of the WHO-AIMS for an initial assessment of their mental health systems - a significant regional effort where 25% of the world population lives [4]. These South East Asia studies, which had implications on a quarter of the World population, only reported data obtained using the WHO-AIMS.

In the African setting, there have been calls for action to develop appropriate polices, efforts to change community attitudes towards mental illness, provision and delivery of health and social services and access to medication and community care in Nigeria and Ethiopia [5], Nigeria [6] South Africa [7] and Uganda [8]. These studies in Africa were not based specifically on the WHO-AIMS.

Kenya has observed similar challenges noted in Brazil, South East Asia, Nigeria, South Africa, Uganda and Ethiopia [9]. However, these Kenyan challenges can be understood in a historical perspective. The Mental Health Act of 1989, referred to as the Act, allowed any hospital in Kenya to admit people with any mental illness and therefore placed emphasis on inpatients [10]. The Act offered some protection for inpatients regarding ill-treatment in hospitals, administration of their estates and examination of females. However, it did not address several human rights of the patients, namely; the patients’ right to information, consent to treatment, and confidentiality, nor did it address the conditions in mental health facilities, or provide for counseling, psychotherapy and rehabilitation services [11]. The Act had other shortcomings, such as limited promotion of community mental services at the primary care level and a lack of distinction between mental illness and mental disabilities [10]. There have been efforts since 2014 to revise the Act; however, as of December 2019, the Bill has not yet been enacted into law, though the draft has significantly improved over the last five years. Currently, it is undergoing a process of public participation to take into account the perspective of all stakeholders including people with mental illness and their families, as provided for by the new Constitution.

Even before the Mental Health Act comes into operation, Kenya has several fallbacks. The most important of these is the Constitution which was promulgated in 2010 [12]. It provides for comprehensive health services, including mental health, as a human rights entitlement [10]. Kenya is also a signatory to international rights conventions which provides state protection of the human and legal rights of people with mental illness and disabilities, their property, and their treatment [13–15]. Kenya has adopted the World Health Organization’s Global Mental Health Action Plan 2013–2020. The objectives of this Action Plan are to ensure effective leadership and governance of mental health services, to provide mental and social care services in community-based settings, to implement strategies for the promotion of mental health and the prevention of mental ill-health, and to strengthen information systems and research in mental health [16]. It seeks to bring the state sector, the private sector, and civil society together in developing policies aimed at improving mental health services, preventing mental illness, and promoting recovery [17]. Equally important is the WHO Mental Health Gap Action Programme Intervention Guidelines (mhGAP-IG) [18] of which Kenya is a signatory. It aims to help address the disparity in mental health care between HIC and LMIC. The package advocates for human resources development, increased financing and effective budgeting, advocacy such as stigma reduction, a community-based approach, improvement of health literacy and multi-disciplinary stakeholders such as formal and informal service providers, enhanced technology information system development, and monitoring and evaluation [9]. The Kenya Mental Health Policy 2015–2030 [19] endeavors to ensure significant reduction in the overall ill-health in Kenya in line with the country’s vision 2030 and the Kenyan Constitution. It provides the framework for interventions to secure mental health systems reforms in Kenya. This is in line with the Constitution of Kenya 2010, which provides for the right to health including mental health, Vision 2030 [20] that projects what Kenya should be able to achieve by the year 2030, and the Kenya Health Policy (2012–2030) [21]. The consequences of poor prioritization of mental health systems has recently attracted the attention of the international media [22].

However, in order to develop an informed mental health system for Kenya and any other country, there is need for evidence that informs the development at baseline and for monitoring and evaluation. The WHO-AIMS was meant to systematically generate that evidence in a reproduce able manner. There has been some effort in Kenya to address mental health systems using the WHO-AIMS [23]. This was a pilot study involving four key stakeholders in two facilities (a public and a private) in Kilifi County at the North Coast of the Indian Ocean. It used the brief version of WHO-AIMS and drew from Kilifi’s health and demographic surveillance system. It found that: policy and legislative framework was based on the only operational but outdated 1989 Mental Health Act, only three outpatients facilities were available in the whole county; no voluntary admission as provided for by the 1989 Act, no documented information on primary health care doctors and nurses who had received at least two days of training in mental health, nurses and non-doctors/non-nurses primary health care workers were not allowed to prescribe psychotropic medications; there were 11.1 per 100,000 population professionals working in public mental health facilities, no psychiatrists and psychologists and 0.2 nurses and social worker per 100,000 population and 0.4 occupational therapists and other health or mental health workers. It also found no continuing education courses in mental health for the staff in the county, no consumer associations or family associations in Kilifi, and no data on monitoring and evaluation. Our study seeks to improve on the effort of the Kilifi study by using the full WHO-AIMS instrument, as well as including more facilities and a wider spectrum of stakeholders. It was intended to inform a program entitled “Multisectoral Stakeholder TEAM Approach To Scale-Up Community Mental Health in Kenya – Building on Locally Generated Evidence and Lessons Learned (TEAM)”. We decided to conduct a baseline study in the existing health system in Makueni County as an entry point for dialogue with the Government of Makueni County and the various stakeholders as a preliminary step towards the implementation of the mhGAP-IG. The process of implementing that program has been documented [24] and some of the outcomes have been published [25–28].

The timing and context of this study, before and after, can be understood in the following chronological order: (1) The study took place approximately four years after the devolvement of health services in Kenya to the county level and the abolishment of the earlier system of two separate ministries i.e. Ministry of Health and Ministry of Public Health and still there was no functional and informed mental health system; (2) The WHO-AIMS study took place between beginning of October 2015 and end of February 2016; (3) The implementation of mhGAP-IG was done in the remaining months of 2016; (4) Data collection and analysis were continuous, followed with publications.

The overall objective of this study was therefore to use the WHO-AIMS prescribed format to establish a baseline analysis for mental health system in Makueni County for future monitoring and evaluation of mental health systems development. To achieve this overall objective, we sought to answer the following research questions: (1) What is the current state of mental health system in Makueni County at the time of this study? (2) What gaps and barriers stood between the current state and a potentially functional mental health system? To answer the questions, we had the following specific aims: (1) To provide a baseline for future monitoring and evaluation of any intervention for the development of a functional mental health system in Makueni County. (2) To identify gaps and barriers in the mental health system; and (3) To make evidence-based recommendations for the development of mental health system in Makueni County.

## Methods

### Study area, population and facilities

This study was conducted in Makueni County, one of the 47 counties in Kenya. It is located about 250 km south-east of the capital city, Nairobi. Makueni County lies astride the Nairobi-Mombasa (the port city) highway. It has a population of approximately one million people, of which 55.8% are under 20 years of age, and is inhabited mainly by the Kamba ethnic community. Its capital is Wote, an urban area with a population of 56,419, of which only 5542 have their homes within the township while the rest have homes outside the township. An arid to semi-arid area, its economy is mainly subsistence farming with 65% of the population living on less than one US dollar per day [29]. The average distance to the nearest health facility is six kilometres [30]. The leading medical conditions are malaria, gastro-intestinal and respiratory tract infections, trauma-related morbidity linked to traffic accidents on the Mombasa-Nairobi highway and occasional natural calamities [31].

Healthcare facilities in Kenya operate in six levels: level 1 – community, level 2 – dispensaries, level 3- health centres, level 4 - sub-county hospitals, level 5 - county referral hospitals, and level 6 - national referral hospitals [32]. Level 1 operates at the community level engaging with individual households and families within the village. Levels 2 and 3 provide mostly promotive and preventive care and sometimes curative services. Levels 4 to 6 address curative and rehabilitative services and to some extent promotive and preventive activities [32]. In consultation with Makueni County Ministry of Health we identified several facilities which are considered to be models for their respective levels. They identified two out of 113 dispensaries, three out of 21 health centres, four out of six sub-county hospitals, and the only county referral hospital.

The WHO-AIMS protocol published in 2005 [33] may not have foreseen this development of devolution. Therefore, for the purposes of this study the term “country” was substituted with “Makueni County”. The respondents were made to understand that their responses were on Makueni County as it stood at the time of the study.

### Study design

The most important aspect of this study was extraction of data relevant to mental health from all available records on topics that have been identified for inclusion by WHO-AIMS. We used all available medical records, including the MOH DHIS, outpatient and inpatient records for the period between January to February 2016. Most of these records were manual, and hence the need for data extraction forms. We used the prescribed template by WHO-AIMS and data extraction forms attached in the supplementary file. The second aspect was interviews of pre-identified persons (described below under research participants) using the WHO-AIMS prescribed format with the specific aim of validating the data already collected.

### Study instrument - the WHO-AIMS

WHO-AIMS instrument was developed by the World Health Organization (WHO) for use in LMICs [33].The development of the WHO-AIMS was interactive and involved experts from LMICs and HIC to confirm clarity, content, validity and feasibility of the WHO-AIMS and also included field pilot trial. It aimed to help LMIC to establish the status of their mental health system at baseline and subsequent follow up on various domains, namely: (1) policy and legislative framework, (2) Organization and integration of mental health services, (3) mental health in primary care, (4) human resources, (5) public information and links with other sectors, and (6) monitoring and research. The WHO-AIMS was developed as a guide, not a measure, and thus has no psychometric properties. It is meant for evaluating a program and not for collecting quantitative data from individuals for statistical analysis. The WHO-AIMS is primarily used to guide, in a standardized way, for extraction of information on mental health from records [33, 34]. Each of the domains is divided into facets and each facet is divided into individual items. The tool has 28 facets and 155 items in total. The tool was used to extract data from the District Health Information System (DHIS) [34] and county official medical records, using data extraction forms. Further, WHO-AIMS provides for key informant interviews (KIIs), purposely to clarify on the information extracted from the records. The themes for interviews are linked to each of the six domains of the WHO-AIMS, where the interviewer would ask the interviewee for their comments on each of the domains. The interviewers would then follow-up with questions for clarification on the response given, until both interviewer and interviewee agreed on what they had covered in the particular thematic area and that a saturation point had been achieved. The interviews were concluded when consensus was achieved using the process prescribed by the WHO-AIMS.

The following are sample illustrations of the KII interviews with various respondents. “*Please tell us about the organization of mental health services in Makueni County”; “Do you have a county mental health authority/body/committee? If yes, what are the functions of the committee*?”; “*How many mental health specialists (doctors/ nurses) do you have in the county?”*

It is to be noted that the emphasis of the questions was not during the process, but after a consensus on what was the most appropriate conclusion from the process of the questions, answers and clarifications.

### Study participants

#### Representation of different offices held by the respondents

To obtain good will from the highest political and executive office, we briefed the Governor on the purpose of our study. We then held a consultative meeting with the Makueni Department of Health through the County Chief Officer of Health on the purpose of the study. We shared with them the ethical clearance certificate and obtained their permission for the study. We agreed that the following would be approached for KIIs as representatives of the official positions they held and therefore, expected to be knowledgeable on health issues from the perspectives of the offices that they led: -

(1) Medical Services, (2) Nursing Services, (3) Health Promotion and Prevention, (4) Commodities, (5) Planning, (6) Education, (7) The chief accountant in the department of health services, (8) The focal person in charge of community health strategy, (9) The county health records officer, (10) The prison warder in-charge of the Government of Kenya prison in Makueni County and (11) The Head of Department of County Social Services (N1 = 11).
The nurse or clinical officer in charge of each the dispensaries and health centres that had been identified for inclusion i.e. (i) two dispensaries (*n* = 2); (ii) Three health centres (*n* = 3) (N2 = 5)
Doctors in-charge of four sub-county hospitals and one county hospital (N3 = 5)
Others: (i) Clinical officer in charge of medical services at the local prison (*n* = 1); (ii) a person with disability (n = 1); (iii) Community officer in charge of disabilities (n = 1); (iv) A counsellor trained on HIV counselling (n = 1) (N4 = 4). Grand total = N1 + N2 + N3 + N4 = 25.

#### Personalized approach to the different respondents

We approached the identified persons in-charge of the various offices to explain to them the nature of the study, shared with them the research clearance and obtained their informed consent to participate. The interviews took place in their own offices at a pre-arranged mutually convenient time.

The KIIs were interviewed so as to respond to all the questions in the predesigned interview schedules generated from the WHO-AIMS and aimed to clarify data extracted from the DHIS. The responses were recorded using paper and pencil.

### Data analysis

The WHO-AIMS provides a standardized information capture template so as to ensure all responses from different countries and different times in a particular study site are comparable for purposes of monitoring and evaluation over time. We, therefore, had to use the recommendations of WHO-AIMS tool. WHO-AIMS is unique and has its own prescribed objectives for the KII from relevant stakeholders– to expound on and validate the data obtained from the records. The narratives from the relevant respondents are meant to give a contextual understanding of the information collected via the WHO-AIMS guide.

We did not seek their own opinion on mental health, but their validation of the information from records, i.e. the focus was on the status of the mental health system and how this information could be used for future monitoring and evaluation of the system and not about changing opinions on mental health by individuals [33]. It is therefore to be noted that we did not conduct in-depth interviews for personal opinions, which is in line with the WHO-AIMS guidelines. We captured illustrative narratives from the different interviews as they clarified the information extracted from the records. If the respondents concurred with the information from the records, then there was not much to be discussed. Data collected from the records was entered on the data entry excel sheets prescribed by WHO-AIMS. We then calculated frequencies and proportions which were summarized in figures and a flow chart, where appropriate.

## Results

In presenting the results, we did not follow the sequence of domains as they appear in the WHO-AIMS. Instead, we rearranged the results in what we considered flows best and indicated to which domain they belong.

### Mental health in primary health care (domain 3)

Makueni County has 142 functional public health facilities consisting of eight hospitals, 21 health centers, and 113 dispensaries. All the clinical personnel in the facilities have foundational knowledge on mental health through course modules on psychiatry taken during their training. Of these facilities, five had previously participated in a training on providing routine screening and intervention for substance use disorders. Staff from the rest of the facilities had not received any additional training on mental health in the last two years, except for the two psychiatric nurses who had attended a psychiatric conference in 2014. There were no specific assessments, or management protocols. One of the doctors noted, “*There are no specific guidelines/protocols. The guidelines used are the ones that were learnt in school, and this is dependent on individual practice.*”

In summary, we identified the following gaps: (1) no specific assessment, management protocols or guidelines for psychiatric care, (2) limited exposure to updates through workshops and conferences among the health workers, (3) no facility-based Continuing Medical Education (CME) offered on mental health except in the two level four hospitals where mental health services were provided by psychiatric nurses, (4) no mentorship and supervisory support for mental health except in two facilities that were run by psychiatric nurses, (5) only eight psychiatric nurses, most of whom are performing general duties for the whole county, (6) no engagement with traditional healers and faith-based healers except occasional health talks regarding mental health issues during religious gatherings. The limited capacity building was associated with low case identification rates for mental disorders in primary care facilities. Health workers also cited limited mentorship and supervisory support for mental health as a sign of the overall poor attention to mental health. Understaffing was also identified as an impediment to delivery of mental health interventions.

One nurse reported, *“We [nurses] fear confronting the [psychiatric] patient and when we see that this is not our case, we refer to the right people”*. Regarding prescriptions, the Chief Officer for Health at the county level noted that nurses are allowed to prescribe medications but with restrictions: “*primary health care nurses are allowed to prescribe but with restrictions (e.g. they are not allowed to initiate prescription but are allowed to continue prescription, or they are allowed to prescribe in emergencies only; they are allowed to hand-out medicines but are formally not allowed to prescribe).”*

The psychiatric nurses reported that they had only engaged non-clinical teams in 10% of the cases they managed. Most times, the interaction between the psychiatric nurse and the informal health workers involved church leaders and teachers as was reported by one psychiatric nurse: *“I do behavioral change communication in my church; I give lectures on how to identify those who have problems (mental and substance use) at an early stage. We also do health talks through school health programs”.*

### Human resources (domain 4)

**Fig. 1:**
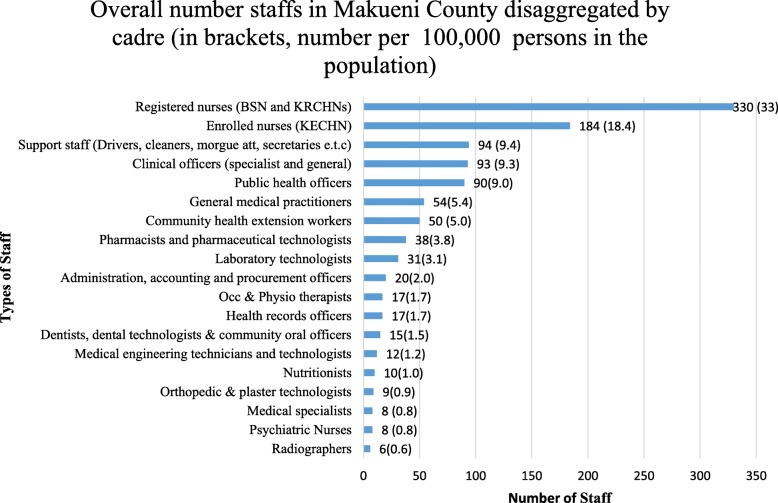
The place of mental health specialist in relation to overall number staffs in Makueni County Disaggregated by Cadre (in Brackets, Number per 100,000 persons in the population)

There was no psychiatrist or clinical psychologist in the entire county. Two out of the eight psychiatric nurses conducted only a one-day psychiatric clinic every week. The overall staffing levels in Makueni County, disaggregated by cadre are illustrated in Fig. 1. There is no specific training for health workers and counsellors on mental health. Instead, they rely on the counselling they received for other illnesses. One of the counsellors noted as follows: “*with the advancement in treatments on HIV/AIDS together with counseling services, most providers have the counseling skills which they use to counsel patients with mental disorders”.* The number of psychiatric nurses (*n* = 8) was second from last in terms of numbers. Due to this shortage, the two psychiatric nurses carry out all the duties in the clinic including managing the stock as alluded by one of the nurses, *“because of the huge shortage, the nurse goes with some tins of necessary drugs and manages the stock himself and this results to unprecedented stock out and pilferages.”*

### Organizational integration of mental health services (domain 2)

The total bed capacity was 616, spread across seven public hospitals, 21 health centres, 113 dispensaries, 11 private facilities, and no designated psychiatric units. There were also no psychiatric beds. Patients requiring psychiatric inpatient services were referred to Machakos Level 5 Hospital in a neighbouring county with sometimes only one psychiatrist and more often none*. “There is no mental inpatient units in Makueni County. Mental cases that are mild are treated in the general wards where patients with other ailments are attended to. There are no specific beds in the wards where mental patients are attended to. When a patient presents with severe mental illnesses, they are referred to Machakos referral hospital or to Mathare Mental hospital in Nairobi,”* indicated one of the psychiatric nurses.

**Fig. 2:**
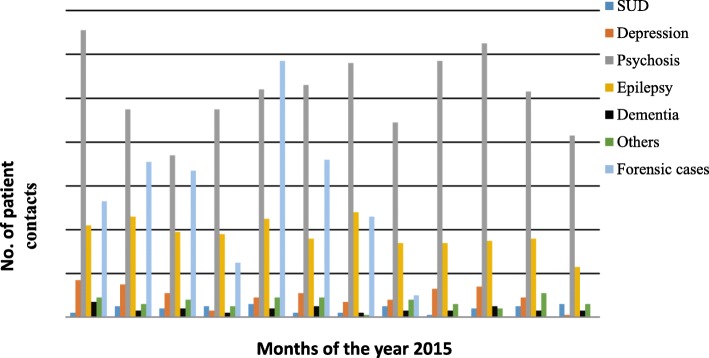
Patterns of clinical diagnoses of mental disorder (2015) at Makueni County at the two health facilities that received mental health training prior to 2015

There were two outpatient mental health clinics open one day a week, which are operated by psychiatric nurses at the Makueni County referral hospital and in one of the participating level 4 hospitals. The number of outpatients seen at the two outpatient psychiatric clinics between January and December, 2015 are illustrated in Fig. 2.

All forensic cases, including mild cases, in need of inpatient care were referred to Machakos Level 5 Hospital. Statistics on the number of patients referred to the hospital due to mental health problems are not available as the DHIS only recorded outpatient numbers.

Makueni County did not have any active rehabilitation facilities for psychiatric cases. However, there were four schools with special units for children with learning disabilities.

Outreach mental health activities were conducted by a psychiatric nurse for free as of 2011 and involved only 13 healthcare facilities located around the County referral hospital. The turnaround time for the psychiatric nurse to return to the same outreach facility was 3 months.

Overall in 2015, a total of 2352 and 1748 contacts (divided by month in Fig. 2) were reported at the DHIS as mental disorders and epileptic disorders respectively. Ten percent of the contacts were made by the psychiatric nurses through outreach clinics.

At the various levels of healthcare facilities, there were costs associated with the specific purpose of the visit. Those who went to dispensaries and health centres were required to bring their own notebooks to document their medical records, which they went home with. For all services, including medication received at these healthcare facilities, a standard fee of twenty Kenyan Shillings (Kshs) (Kshs.20/− equivalent to 0.20 US Dollar (USD)) was paid. If the medicines were out of stock at the healthcare facility, patients were free to buy them from private pharmacies at a cost.

At levels 4 and 5 healthcare facilities, patients were provided with cards for their medical records which were retained at the healthcare facilities. At these level 4 and 5 healthcare facilities, patients paid a standard fee of two hundred Kenyan shillings (Kshs.200/− i.e. 2 USD) for all services provided, including medication. In this case if the medication required was out of stock, patients were required to make arrangements in order to buy the medications from a private pharmacy of their choice. Further, at levels 4 and 5 there was a waiver system in case the patients could not afford the Kshs.200/−, but on recommendation of a social worker. However, this system did not exist at the lower levels as no social workers are deployed at these levels.

**Fig. 3:**
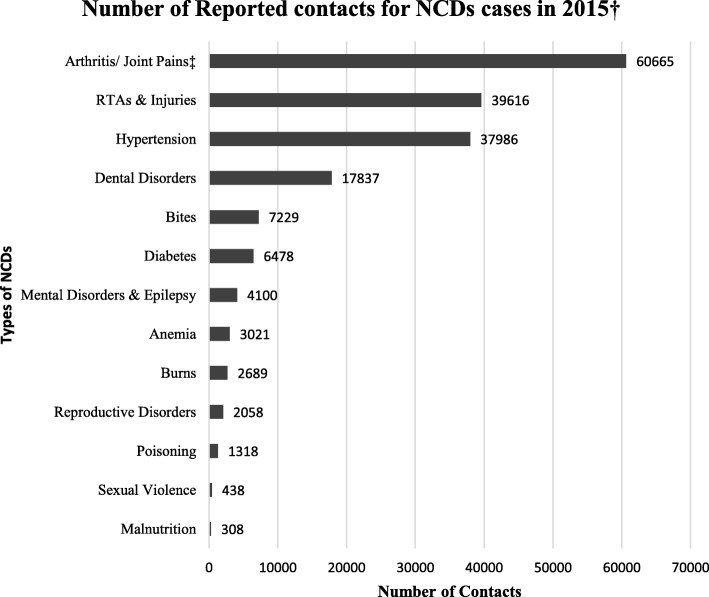
Prevalance of clinical diagnosis of mental disroders and epilepsy in relation to other reported cases for Non-Communicable Diseases in Makueni County in 2015

In the actual reporting to the county information system, which is what is transmitted to the national health system as part of the overall health system information, mental disorders and epilepsy were grouped into one category that also includes substance use disorder as summarized in Fig. 3. (*Highlighted for quick reference*).

Makueni County did not have a department of mental health and lacked representation at the departmental heads’ meetings where matters related to mental health policy, practice and administration would be discussed at the County level.

### Monitoring mental health services (domain 6)

There was no routine collection and reporting of key data on mental illnesses and there was no formally defined list of individual data items that ought to be collected by all mental health facilities. The single available data collection tool only captured mental disorders in aggregated form apart from epilepsy. Thus, specific psychiatric morbidity statistics were not available at the county level and so, none was forwarded to the national level.

### Formal linkages, coordination and collaboration with other sectors (domain 5)

There were no formal collaborative programs addressing issues of persons with mental health problems for teachers, schools, the police, and the prison services.

The clinical officer running the prison health services in the county had not received additional training or updates on mental health. The prison warden also reported that they had challenges handling mental health cases as they had not received formal training. The only non-governmental organization (NGO) working on mental health was the Africa Mental Health Foundation (AMHF), now renamed Africa Mental Health Research and Training Foundation. The director for preventive services summarized the situation by noting, “*There does not exist any coordinating bodies that oversee public education and awareness campaigns on mental health in Makueni County apart from an NGO called Africa Mental health Foundation. The only NGO that has spearheaded mental health campaigns is the Africa Mental Health Foundation. The general health awareness has been made vibrant this year 2015. Initially, it was not taken care of. When gatherings or other social functions are taking place, NGO (AMHF) get involved and that is how they channel their information to the community. Africa Mental health Foundation deals with all sub groups in the general population. Other Institutions or NGOs focus on general health and does not narrow down their mandates to Mental Health. An example is the APHIA Plus which is well known in spearheading general health in the County. There is also the Kenya Psychological Counseling Association that was recently launched (2-3 months old) but it has not yet been operationalized”.*

On existence of legislative provisions concerning a legal obligation for employers to hire a certain percentage of employees that are disabled, interviews with one of the disabled persons-who is an Albino and a teacher by profession, revealed that there is a huge discrimination against disabled persons in the County. “*As PLWD (people living with disability), we are perceived to be non-performers and so they don’t employ us. It took me about 3 years to secure a job as a teacher because of my disability and not my inability*”. The County Director for persons with disabilities in Makueni County reported that they usually draft recommendation letters to PLWD people to serve as a backup on their CV; however, clearly it was stated that a great deal of misconceptions regarding PLWD and employment exist. There are no legislative or financial provisions concerning priority in state housing and in subsidized housing schemes for people with severe mental disorders. “*This only targets severe cases. For instance, people who are totally paralyzed are catered for by the Social Protection Program and they are given Ksh. 2000 (20USD) per month to take care of housing and food*” noted the director.

### Mental health policy (domain 1)

There was no operational and specific policy on mental health at the national and county level at the time of the study. The only one available was on general health. The chief officer for health noted that, “There is a National health policy 2013 that is being used. There is also a draft that covers all the sectors of health-County strategic plan.” Makueni came up with a 2013–2015 plan. In the County draft, there is an indicator variable for mental health but with no target. It states: “Need to establish one mental health in the County, construct a mental unit”. However, Makueni County had some fall backs: (1) Mental health activities were governed by the Mental Health Act Chapter 248 of 1989 [35], which defined healthcare facilities that could admit people with mental illness and provided for voluntary admission for a patient with a psychiatric disorder. (2) In article 43 of the constitution of Kenya, Kenyans are guaranteed access to basic rights in which the right to health and treatment are clearly documented. Additionally, part 2 of the fourth schedule of the Kenyan constitution defines the provision of mental health services as a main function of the county government [36]. (3) The WHO Mental Health Action Plan 2013–2020 [37]. (4) Makueni County developed a County Integrated Development Plan (CIDP) 2013–2017 [38], which amongst other things, identifies the provision of emergency psychosocial support and counselling as a key flagship project for the county. This project was initiated under the guidance of the First Lady of Makueni County. However, there is still the need to contextualize these fall backs in a policy framework which did not exist at the time of the study.

**Fig. 4:**
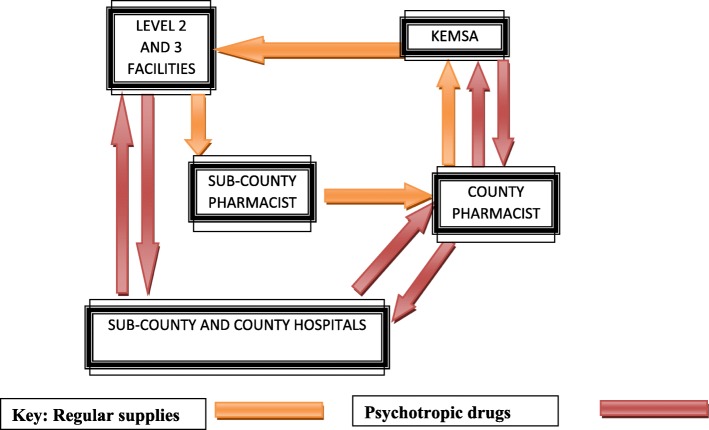
Longer chain to procure psychotropic than non-psychotropic: Stock management supply chain flow chart at Makueni County

Psychotropic Medication: Fig. 4 summarizes the existing drug procurement procedure at the time of study. It indicates that there was a longer procedure for securing psychotropic drugs as opposed to non-psychotropic drugs even for psychotropics on the essential drug list such as diazepam, phenobarbital and chlorpromazine tablets and injectable that were allowed for level 2–3. Stigma affects procurement of medicines. “*Stigma on mental disorders affects procuring of medicines. It is termed as “Dawa ya waenda wazimu“- directly translated to mean “medicines for mad people”, the county pharmacist indicated.*

Non-psychotropic drugs to dispensaries and health centres (level 2 and 3 respectively) did not have to go through sub-county or county hospitals and could be supplied directly from Kenya Medical Supplies Agency (KEMSA) - the national drug store and supplier. However, in the case of psychotropic drugs, supplies could only be from or through the county and sub-county hospitals.

## Discussion

We present an up to date, most detailed study in Kenya on mental health systems using the WHO-AIMS. According to our literature search, using pub-med, with the key terms; WHO-AIMS, Kenya, mental health assessment systems from 2005 (when WHO-AIMS was published) to date (6th December 2019), we could only find one study done in Kenya (reviewed under the literature), which studied only two health facilities one private and one public, interviewed four stakeholders and used a brief version of WHO-AIMS. Our current study used the full version of the WHO-AIMS, had a larger catchment area, interviewed a wider spectrum of informants (*N* = 25), including the law enforcement and representation of all stakeholders in mental health, and studied a representative sample of all levels of health care systems in Makueni County. We wish to point out that at the onset of our discussion, all the respondents, including the head of the services at policy level, pointed gaps and barriers in the mental health system, despite all of them being interviewed independently. It is therefore unlikely that any of them was influenced to give a false positive response or to deny the validity of the data extracted from records.

The most positive point of the findings was that there was a positive inclination to mental health as evidenced by the wide spectrum of documents that advocated for mental health including the Constitution of Kenya. What lacked were operational policies and identification, and maximization of various existing opportunities.

This study showed that at the time of the study, Makueni County had not developed its own policies and structures for implementing mental health. The National Government did not have an operational mental health policy either to guide the County Governments. From the analysis of this baseline survey on records and validated by the respondents, we identified several opportunities that could be optimized in subsequent implementation research. These included: (1) adaptation and adoption of existing county strategic documents to support provision of universal mental health through context appropriate policies, structures and supervision; (2) adaptation and adoption of prescription policies based on the essential drugs list to include levels 2 and 3 facilities to store certain psychotropic drugs, but with training and technical support as suggested by [39, 40]; (3) maximization, mobilization and empowerment of existing human resources to accommodate mental health; (4) integration of mental health in the services for physical conditions using existing health system that currently excludes mental health. For example, arthritis and pains, the most common reported NCD (Fig. 3), largely camouflage the diagnoses of psychiatric disorders [41, 42]. This existing health system relies heavily on task shifting that uses non-specialists health workers who are trained, supported and supervised with the option to refer complicated cases [43–45]. However, these existing health systems largely exclude mental health, further emphasizing the need to integrate both systems. At the time of the study, Makueni had 8 psychiatric nurses, most of whom deployed to perform administrative or other non-psychiatric duties, which illustrates their relative unavailability to provide quality mental health services full-time. As a result, there is a need for task shifting in mental health services to include non-psychiatric users and clinical officers.

Integrated services for both mental health and physical condition services will be cost-effective as mental disorders are identified and managed early, and preventive programs can be embedded within the services, as has been observed elsewhere [39]; (5) adaptation and adoption of the universally used mental health gap action program intervention guidelines for evidence based [37, 46] (6) Inclusion of the already existing and relevant stakeholders in a collaborative effort on mental health, which include healthcare providers, service users and policy makers, judicial, correctional, educational, social and family-oriented services. The optimisation of opportunity #6 could potentially lead to challenges of demand versus service provision. The creation of demand for services or health-seeking behaviour for mental health services must be accompanied by the development of capacity to accommodate for the increased demand, thus emphasizing the need for enhanced task shifting and task sharing for mental health services.

On the other hand, enhanced capacity for the primary health workers to identify and manage mental disorders has the potential to avoid the same persons coming back for services because they had not been properly diagnosed and managed, creating a revolving door phenomenon. It will instead potentially reduce demand for services due to proper diagnoses and management, get better results, and enhance the morale of the service providers [25, 40, 47]. Makueni County did not have a data-capture mechanism that disaggregated mental health data in terms of specific conditions. Such data would be useful to inform prioritization of mental health resource allocations and interventions.

### The way forward

The most basic finding of this study is the scarcity of a functional mental health system in our study area. However, this finding provides a baseline for action-oriented approach to the development of a mental health system. It is our opinion that this can occur only if there is a legal framework in the form of a mental health policy, supported by Acts of parliament at National and County levels aiming to operationalize policies and practices that accommodate mental health in the following areas; capacity building in human resources in mental through training of new staff and retraining of already available staff through continuous medical education (CME); integration of mental health into the already existing services at community primary health care and facility levels including corrective/rehabilitation services in prisons, which calls for development of an integrated health information systems to include mental health; being responsive to the human rights of people with mental illness and disabilities. Having the baseline in place, there is need for periodic application of the WHO-AIMS in order to monitor and evaluate any changing in patterns in the development of the mental health system in Makueni County in an objective, documentable and reliable way. Given the feasibility of this study in Makueni County, there is the potential that it can be repeated in the rest of the counties in Kenya and other similar LMICs specifically using the WHO-AIMS instrument for purposes of standardized baselines and follow-ups. In order to achieve all the above, there is a need for collaborative efforts by different disciplines and different stakeholders from policy makers to service providers with a focus on mental health.

## Conclusions

1. The findings of this study are comparable to those found in other LMICs summarized under the introduction, pointing out the lack of functional mental health systems despite different health systems.
2. We have achieved our general objective of establishing a contextualized baseline on mental health system as at the time of the study, at Makueni County in Kenya, using the WHO-AIMS.
3. We have answered our two research questions: We established the mental health system status at the time of the study and identified the gaps and the barriers that stood between the then current status and a potentially functional mental health system.
4. In achieving the overall objective and answering the research questions, we achieved our three specific aims: (1) We established a baseline for future monitoring and evaluation of any intervention for the development of a functional mental health system in Makueni County. (2) We identified the then gaps and barriers in the mental health system; and (3) We made evidence-based recommendations for the development of mental health system in Makueni County.

### Limitations

1. An apparent limitation of this study is part and parcel of the limitations of the WHO-AIMS, which has no documented psychometric properties as detailed under Methodology (study instrument) because it was primarily designed for program evaluation, and not the socio-demographics of the interviewees. It is therefore not possible to provide psychometric properties of the instrument, nor collect data on the socio-demographics of the interviewees. Further, we interviewed representatives of offices in their official capacities regardless of their socio-demographic characteristics, for the sole purpose of validating information extracted from records.
2. These results may not be generalized to all the 47 counties in Kenya. However, there are mitigations to this limitation: (i) Nearly all counties in Kenya have similar health systems governance and resources except a few urban populations that benefit from a high concentration of resources, such as almost all 100 or so psychiatrists and nearly all mental health specialists for the approximate population of 45 million Kenyans; (ii) this study demonstrates the feasibility of carrying out context appropriate studies in other similar settings using the WHO-AIMS.
3. We purposively sampled the facilities and the key informants to participate in this study; therefore, all facilities in the county were not included. In mitigation, a combined team from AMHRTF and Makueni County’s Department of Health ensured that all levels of healthcare in the county were represented in the sample.
4. All Key Informant Interviews were conducted on the most senior staff at policy level and heads of various services at different levels of facilities who knew the system well by virtue of their designated official status, but did not include consumers of services who could have their own thoughts, although they may not have been conversant with the health system structures. Indeed, service consumers are not specifically provided for in WHO-AIMS. However, they could be considered in future versions of WHO-AIMS.

## Data Availability

The data that supports the findings of this study are available from the corresponding author upon request.

## Abbreviations

AMHRTF: Africa Mental Health Research and Training Foundation
CIDP: County Integrated Development Plan
DoHS: Department of Health Services
KEMSA: Kenya Medical Supplies Agency
MhGAP-IG: The WHO Mental Health Gap Action Programme Intervention Guidelines
MOH: Ministry of Health
WHO-AIMS: World Health Organization Assessments Instrument for Mental Health Systems
